# Supplementary material for: Chemical Transformations in Proto-Cytoplasmic Media. Phosphorus Coupling in the Silica Hydrogel Phase
Source: Life (Basel). 2017 Nov 19;7(4):45. doi: 10.3390/life7040045 (PMC5745558; doi:10.3390/life7040045)
Supplement: Supplementary file 1 [file life-07-00045-s001.pdf]

life

Supplementary Materials: Chemical Transformations in Proto-cytoplasmic Media. Phosphorus Coupling in the Silica Hydrogel Phase

Ian B. Gorrell, Timothy W. Henderson, Kamal Albdeery, Philip M. Savage and Terence P. Kee *

1. Silica hydrogel nomenclature

We have selected to name our gel formulations as either 0.5 M, 1.0 M or 1.4 M wrt SiO_2 on the basis of the following calculations:

The silicate solution that we use is graded at ≤ 27% SiO_2. Thus, this translates to ≤ 27 g in 100 g of solution; which is 27/60.08 =0.45 moles. Therefore

As the density of the silicate solution we use is 1.39 g/mL, therefore 100 g of silicate solution constitutes 72 mL of solution.

Consequently, a solution which contains 0.45 moles in 72 mL of solution translates to a concentration of 6 M.

Thus, as our SHG formulations require diluting 1250, 2500 and 3750 μL respectively of this stock solution to 16 mL; dilution factors of 0.078, 0.16 and 0.23 respectively; these three formulations contain silica concentrations at 0.47 M, 0.96 M and 1.38 respectively. We have rounded these figures to the first significant decimal point in the nomenclature.

2. Isohypophosphate preparation

We prepared isohypophosphate using the procedure published elsewhere (Bryant, D.E.; Herschy, B.; Marriott, K.E.R.; Cosgrove, N.E.; Pasek, M.A.; Atlas, Z.D.; Cousins, C.R.; T. P. Kee, T.P. Phosphate Activation *via* Reduced Oxidation State Phosphorus (P). Mild Routes to Condensed-P Energy Currency Molecules, *Life*, **2013**, *3*, 386-402). The material used in experiments herein was a mixture of isohypophosphate [PPi(III-V); $H_2P_2O_6^{2-}$] along with the starting materials Pi(III) [$H_2PO_3^-$] and Pi(V) [$H_2PO_4^-$] with composition determined by ^{31}P-NMR spectroscopy, see Figure S1 (NMR setting as described in the manuscript).

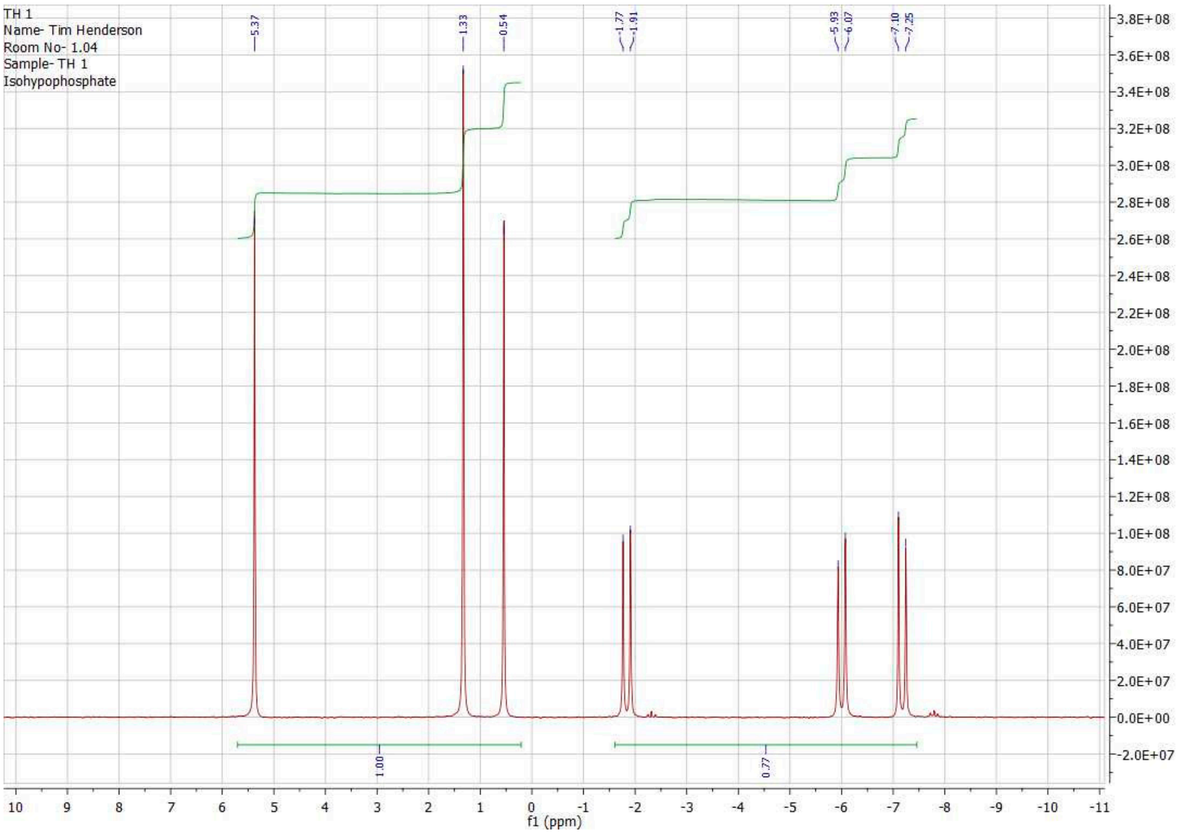

Figure S1. ^{31}P-NMR spectrum of a mixture of isohypophoshate [PPi(III-V)], Pi(III) and Pi(V) in the proportions [PPi(III-V)]-[Pi(III)/Pi(V)] in the relative proportions: 44%–56%.

3. Representative ^{31}P-NMR spectrum of a Pi-coupling preparation in silica hydrogels

In Figure S2 is shown a representative ^{31}P-NMR spectrum acquired under the conditions indicated in the manuscript (5 mm tubes on a Bruker Avance III 300 operating at 121.495 MHz for ^{31}P, using D_2O or a D_2O-containing capillary to provide the lock signal. NMR analyses were performed with both a 3 s pulse delay and in a gated ^{1}H coupled mode in order to limit any nuclear Overhauser effects which could compromise integration measurements. In each case, 320 transients were obtained). In this case, the spectrum is of the reaction G18 (Table 2 in the manuscript) a SHG (1.5 M silica formulation) reaction containing PPi(III-V), Fe(II) deposited on the surface of the SHG and H_2O_2 included in the acid phase. It demonstrates the presence of PPi(V) in 9.4% of total Pi present.

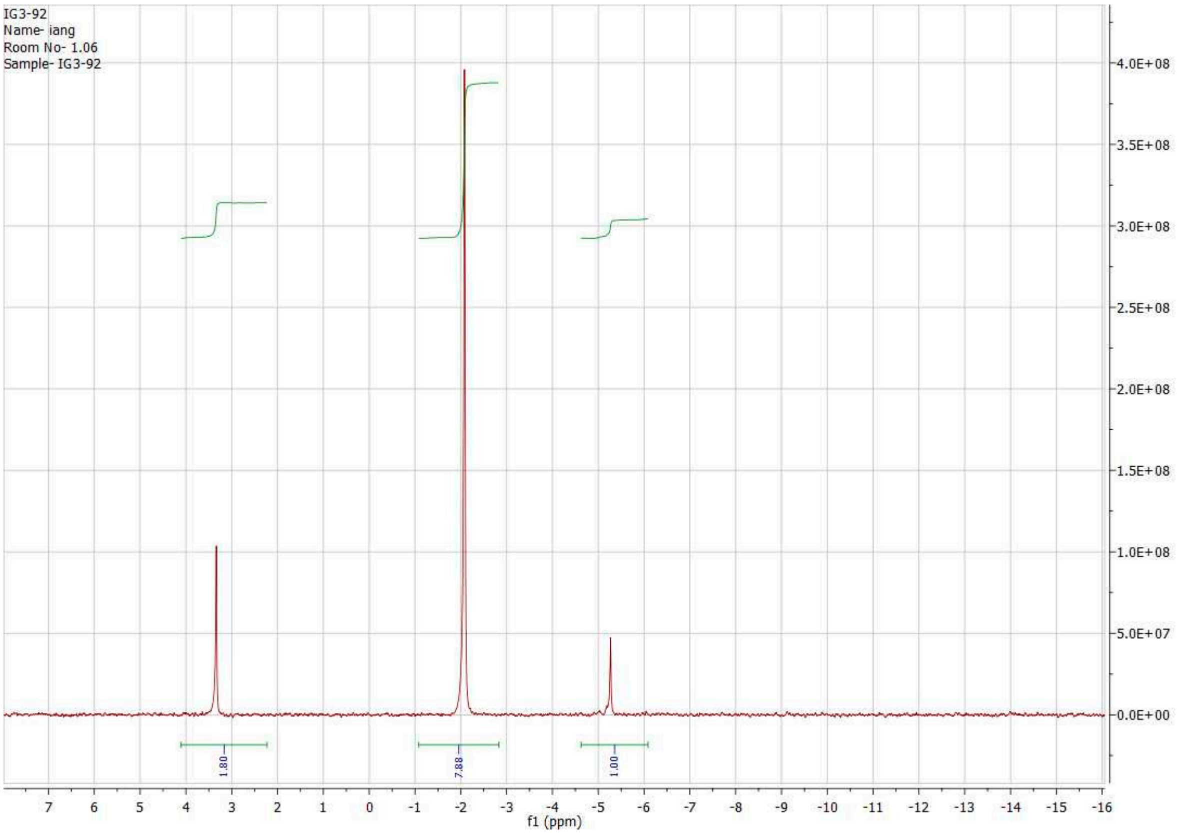

Figure S2. ^{31}P-NMR spectrum of reaction G18 demonstrating the presence of PPi(V) in 9.4% of total Pi (signal at δ-5.3 ppm).

4. BET surface area measurements

Noted in the narrative that we find that overall BET surface areas of the silica matrices increase from *ca* 190 m^2/g to *ca* 405 m^2/g post-dialysis. The analytical data are collected in the attached excel files.
